# Protocol to phenotype and quantify mycobacteria-specific myeloid cells from human airways by mass cytometry

**DOI:** 10.1016/j.xpro.2024.103463

**Published:** 2024-12-13

**Authors:** Agano Kiravu, Virgine Rozot, Lauren Cruywagen, Andrea Gutschmidt, Nelita DuPlessis, Elisa Nemes, the POI-BAL study group, Michele Tameris, Thomas Scriba, Arina Conradie, Fazlin Kafaar, Ilana C. van Rensburg, Gerhard Walzl, Stephanus Malherbe, Ayanda Shabangu, Keren Middelkoop

**Affiliations:** 1 South African Tuberculosis Vaccine Initiative, Institute of Infectious Disease and Molecular Medicine and Division of Immunology, Department of Pathology, University of Cape Town, Cape Town 7925, South Africa; 2 DSI-NRF Centre of Excellence for Biomedical Tuberculosis Research, South African Medical Research Council Centre for Tuberculosis Research, Division of Molecular Biology and Human Genetics, Faculty of Medicine and Health Sciences, Stellenbosch University, Cape Town, South Africa

**Keywords:** Cell culture, Mass Cytometry, Immunology

## Abstract

Alveolar macrophages and other myeloid cells in the human airways are the primary cell types responding to respiratory pathogens. Here, we present a protocol for *in vitro* stimulation of cryopreserved human bronchoalveolar lavage (BAL) cells with mycobacterial antigens for phenotyping and quantifying proinflammatory cytokine responses in myeloid cells by mass cytometry. We demonstrate that the measure of markers of myeloid lineage and function is stable after freezing stained cells thereby allowing for batched analyses and/or machine downtime.

## Highlights

• Functional and phenotypic characterization of human alveolar macrophages
• Steps for thawing and stimulation of cryopreserved bronchoalveolar lavage cells
• Steps for staining of bronchoalveolar lavage cells after antigen stimulation
• Optimized antibody panel for mass cytometry

## Abstract (editor highlights)

Publisher’s note: Undertaking any experimental protocol requires adherence to local institutional guidelines for laboratory safety and ethics.

## Graphical abstract

**Figure:**
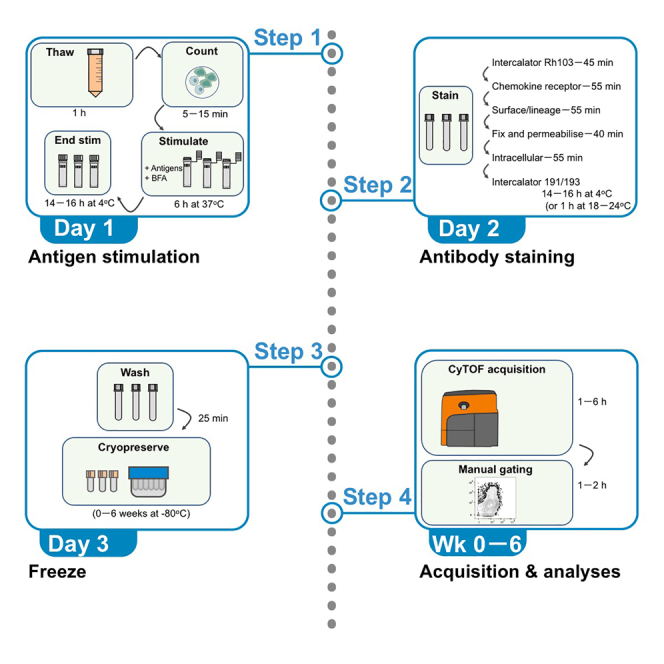

## Before you begin

The following is an optimized protocol to phenotype and quantify mycobacteria-reactive myeloid cells from human BAL by mass cytometry. We provide optimized culture conditions to maximize BAL cell viability, recovery, and cytokine production. Incorporated in this workflow is the flexibility to freeze stained samples for batch analyses. The antibody panel used in this protocol is myeloid focused with 10 antibodies conjugated in-house (see key resources table and reagent preparation for details). Other customized panels can be applied with careful consideration for marker abundance, signal spillover etc.^1^ For optimal panel design, it is recommended to use the web-based interactive panel designer provided by Standard Biotools (Panel Designer v2.0.1)^2^ available on the Standard Biotools website. All antibodies should be titrated to optimize resolution and, where appropriate, validated on other tissues where expression is expected (see troubleshooting section). We recommend including a control sample of peripheral blood mononuclear cells (PBMCs) from a selected donor to monitor batch effects, which can be corrected in downstream analyses. Staining buffers and other reagents used in the protocol should be routinely tested for Barium or other non-specific metal contaminants that can contribute to detector ageing and false positive signal, respectively.^3^ Details on BAL cell collection, initial processing and cryopreservation have been previously described.^4^

Institutional Review Board (IRB) approval for sample collection and adherence to safety protocols for handling potentially infectious broncho-alveolar lavage samples is required.

### Institutional permissions

Written informed consent was obtained from participants providing samples in this study and the protocol was approved by the Institution’s IRB. Study participants were healthy and tested negative for HIV, SARS-CoV2 and *Mycobacterium tuberculosis* (Mtb), however cryopreserved samples were treated as potentially infectious during processing, which occurred in a Biosafety level 2 facility. All personnel working with BAL samples were trained in the appropriate handling and disposal of biological material. Approval for this study was obtained from the University of Cape Town’s Human Research Ethics Committee (ref. 002/2020).

### Reagent preparation

**Timing: 1–6 h**

**Timing: 1 h (for step 1)**

**Timing: 5 min (for step 2)**

**Timing: 6 h (for step 3)**

**Timing: 6 h (for step 4)**

1. Preparation of Mtb lysate antigen.
  This section describes the preparation of working stock of Mtb antigen from lyophilized stock. Working stocks for the assay are frozen until the day of use.
  a. Allow the vial containing lyophilized Mtb proteins (10 mg/vial) to reach 18°C–24°C and add 5 mL of sterile 1 x PBS to make a 2 mg/mL stock.
  b. Vortex to completely dissolve the lyophilized proteins (for approximately 20–30 s).
  c. Prepare a working stock solution at 150 μg/mL in sterile 1x PBS (i.e., add 6.67 mL of sterile PBS to 0.5 mL of the stock solution).
  d. Store in 100 μL aliquots at −80°C until use.
2. Preparation of Bacille Calmette-Guerin (BCG) antigen.
  This section describes the preparation of BCG antigen from vaccine vials. Working stock for the assay is prepared on the day of use.
  a. Under a biosafety cabinet, spray and wipe unopened vial with 70% ethanol.
  b. Spray and wipe 20 mm vial crimper with 70% ethanol before removing vial cap and rubber stopper.
  c. Add 500 μL of stimulation media to achieve a CFU concentration of ∼10 × 10^6^ CFU/mL.
  d. Replace rubber cap, invert vial and vortex for 1 min to ensure resuspension.
3. In-house antibody conjugations.

Antibody conjugates for specifically designed panels may not be commercially available, requiring in-house conjugations specific for Lanthanide and Cadmium isotopes.

***Note:*** Antibody conjugations were performed as described in the MaxPar antibody labeling kit package inserts, referenced in the key resources table, below. In our hands this protocol achieved average recoveries of 54% and 89% for Lanthanide and Cadmium conjugated antibodies respectively (Table S1).

4. Antibody titrations.

Antibody titrations are an essential component of cytometry experiment design to provide the best signal-to-noise ratio, minimize background from nonspecific binding and signal overlap and allow for the optimal detection of low abundance markers. The advantage of mass cytometry is that multiple antibodies can be titrated simultaneously in the same tube as isotope signal overlap is constant and can be predicted (isotope impurities, oxides spread and abundance sensitivity are known).

***Note:*** We performed titration by designing smaller panels of 5–8 markers in 3 carefully designed titration runs taking into consideration isotope signal and the appropriate sample type (Table S2). Additionally, we included core markers such as CD3 for T cells or CD206 for myeloid cells in channels not overlapping with tested markers to assess titrations with a biological readout in a cell-specific manner to evaluate markers on rare populations. Starting at 2 μg of antibody in 100 μL volume, two-fold serial dilutions were prepared over 7 concentrations (see Table S1). Both the staining index and absolute isotope signal was plotted for each marker to determine the lowest titer resulting in the maximum signal to noise ratio.

## Key resources table

**Table:** 

REAGENT or RESOURCE	SOURCE	IDENTIFIER
**Antibodies**		

CD45-Y89 (clone HIB30, 1:100 dilution)	Standard BioTools	Cat# 3089003B
CD206-112Cd (clone 15-2/MMR, 1:200 dilution)	BioLegend	Cat# 321127
CD103-145Nd (clone Ber-ACT8, 1:200 dilution)	BioLegend	Cat# 350202
TNF-146Nd (clone MAb11, 1:100 dilution)	Standard BioTools	Cat# 3146010B
CD33-148Nd (clone WM53, 1:200 dilution)	BioLegend	Cat# 303419
CD56-149Sm (clone NCAM16.2, 1:400 dilution)	Standard BioTools	Cat# 3149021B
CD274-150Nd (clone 29E.2A3, 1:100 dilution)	BioLegend	Cat# 329719
CD14-151Eu (clone M5E2, 1:100 dilution)	Standard BioTools	Cat# 3151009B
IL1ß-152Sm (clone JK1-B1, 1:200 dilution)	BioLegend	Cat# 508201
CD163-154Sm (clone GHI/61, 1:100 dilution)	Standard BioTools	Cat# 3154007B
IL6-156Gd (clone MQ2-13As, 1:200 dilution)	Standard BioTools	Cat# 3156011B
CD169-158Gd (clone 7-239, 1:100 dilution)	Standard BioTools	Cat# 3158027B
CD11c-159Tb (clone Bu15, 1:100 dilution)	Standard BioTools	Cat# 3159001B
CD66-161Dy (clone 5B2, 1:800 dilution)	Invitrogen	Cat# MA117760
CXCR3-163Dy (clone G025H7, 1:100 dilution)	Standard BioTools	Cat# 3163004B
CD19-164Dy (clone SJ25C1, 1:400 dilution)	BioLegend	Cat# 363002
CD40-165Ho (clone 5C3, 1:100 dilution)	BioLegend	Cat# 3165005B
CD11b-167Er (clone ICRF44, 1:100 dilution)	Standard BioTools	Cat# 3167011B
CD32-169Tm (clone FUN2, 1:200 dilution)	Standard BioTools	Cat# 3169020B
CD3-170Er (clone UCHT1, 1:100 dilution)	Standard BioTools	Cat# 3170001B
CCR7-171Yb (clone G043H7, 1:100 dilution)	BioLegend	Cat# 353237
HLA-DR-173Yb (clone TU36, 1:200 dilution)	BioLegend	Cat# 361602
CD89-176Yb (clone A59, 1:100 dilution)	BioLegend	Cat# 354102
CD16-209Bi (clone 3G8, 1:100 dilution)	Standard BioTools	Cat# 3209002B

**Chemicals, peptides, and recombinant proteins**		

RPMI 1640 medium	Gibco	Cat# BE12-702F
Amphotericin B	Gibco	Cat# 15290-026
Penicillin-streptomycin	Gibco	Cat# 15070063
L-glutamine GMP (200 mM)	Gibco	Cat# 25030081
Brefeldin A (BFA)	Sigma-Aldrich	Cat# B7651-5MG
Fetal bovine serum (FBS)	HyClone	Cat# SV30160.03IR
Deoxyribonuclease I from bovine pancreas (DNase I)	Sigma-Aldrich	Cat# D4513-1VL
Dimethyl sulfoxide (DMSO)	Sigma-Aldrich	Cat# D5879
Cell-ID Intercalator-Ir_125 μM	Standard BioTools	Cat# 201192A
Cell-ID Intercalator-Rh_500 μM	Standard BioTools	Cat# 201103A
EQ four element calibration beads	Standard BioTools	Cat# 201078
Maxpar cell staining buffer	Standard BioTools	Cat# 201068
Maxpar Fix and Perm buffer	Standard BioTools	Cat# 201067
Maxpar Perm-S buffer	Standard BioTools	Cat# 201066
Maxpar 5X Fix I buffer	Standard BioTools	Cat# 201065
Trypan blue dye	Gibco	Cat# 15250061
Bond-Breaker TCEP solution, neutral pH∗	Thermo Fisher Scientific	Cat# 77720
HRP-Protector peroxidase stabilizer	Boca Scientific	Cat# 222 050 (50 mL)
Antibody stabilizer PBS (supplement with sodium azide after purchase)	Boca Scientific	Cat# 131 050 (50 mL)
Sodium azide, BioUltra, ≥99.5%^†^	MilliporeSigma	Cat# 71289
Mycobacterium tuberculosis, H37Rv, whole-cell lysate	The following reagent was obtained through BEI Resources, NIAID, NIH: *Mycobacterium tuberculosis*, strain H37Rv, whole-cell lysate, NR-14822	Cat# NR-14822
Lyophilized BCG vaccine	SSI Denmark	N/A
Phosphate-buffered saline	Lonza	Cat# BE17-517Q
Ethylenediaminetetraacetic acid disodium salt solution (EDTA)	Sigma-Aldrich	Cat# E7889
70% ethanol	–	N/A

**Software and algorithms**		

CyTOF Software V6.0	Fluidigm	https://www.fluidigm.com/products-services/software
FlowJo V10.6	Becton Dickinson	N/A
R	N/A	R Core Team (2021)

**Other**		

Maxpar MCP9 antibody labeling kit, 112Cd	Standard BioTools	Cat# 201112A
Maxpar X8 antibody labeling kit, 145Nd	Standard BioTools	Cat# 201145A
Maxpar X8 antibody labeling kit, 148Nd	Standard BioTools	Cat# 201148A
Maxpar X8 antibody labeling kit, 150Nd	Standard BioTools	Cat# 201150A
Maxpar X8 antibody labeling kit, 152Sm	Standard BioTools	Cat# 201152A
Maxpar X8 antibody labeling kit, 161Dy	Standard BioTools	Cat# 201161A
Maxpar X8 antibody labeling kit, 164Dy	Standard BioTools	Cat# 201164A
Maxpar X8 antibody labeling kit, 171Yb	Standard BioTools	Cat# 201171A
Maxpar X8 antibody labeling kit, 173Yb	Standard BioTools	Cat# 201173A
Maxpar X8 antibody labeling kit, 176Yb	Standard BioTools	Cat# 201176A
Amicon Ultra-0.5 mL centrifugal filters Ultracel-3 KDa	Millipore	Cat# UFC500396
Amicon Ultra-0.5 mL centrifugal filters Ultracel-50 KDa	Millipore	Cat# UFC505096
NanoDrop 1000 spectrophotometer	Thermo Fisher Scientific	N/A
Microfuge 22R centrifuge	Beckman Coulter	N/A
Allegra X-12R centrifuge	Beckman Coulter	N/A
Desktop mini centrifuge	N/A	N/A
Class II biosafety cabinet	N/A	N/A
Zeiss Axiostar binocular microscope	Carl Zeiss	N/A
Micro tube 1.5 mL	Sarstedt	Cat# 72.694.106
Corning 500 mL bottle-top vacuum filters with 0.22 μm membrane	Millipore	Cat# CLS430513
Ultrafree-MC centrifugal filter devices with Durapore Membrane	Millipore	Cat# UFC30VV00
Milli-Q 1X water system	Merck	N/A
Falcon 5 mL round bottom polystyrene tube with snap cap	Falcon	Cat# 352054
Falcon 5 mL round bottom polystyrene tube with cell strainer snap cap 35 mm	FlowTubes	Cat# T9005
Screw cap micro tube, 2 mL, sterile	Sarstedt	Cat# 72.694.106
Pipette set (P10, P20, P200, P1000)	Gilson	N/A
Sterile filtered pipette tips (P10, P20, P200, P1000)	N/A	N/A
Individually wrapped filter plugged serological pipettes (10 mL, 25 mL)	Pro Lab Supply	Cat# 9100, 91025
Cryogenic vials with internal thread	SPL Life Sciences	Cat# 43022
Freezing container, Nalgene Mr. Frosty	Sigma-Aldrich	Cat# C1562-1EA
20 mm vial crimper	N/A	N/A

**CRITICAL:** ∗^†^Precautions should be taken when handling TCEP and sodium azide. Avoid exposure to eyes, skin, or clothing. Wear protective gloves, protective clothing, eye protection, face protection and avoid release into the environment. Wash hands thoroughly with soap and water after exposure to skin and handling.

## Materials and equipment

**Table:** Thawing media

Reagent	Final concentration	Amount
RPMI 1640 media	88%	44 mL
Heat inactivated FBS	10%	5 mL
Penicillin-Streptomycin	1% (50 U/mL)	0.5 mL
Fungizone (Amphotericin B)	1% (2.5 μg/mL)	0.5 mL
**Total**		**50 mL**

Filter through 0.22 μm membrane filter. Store at 4°C for up to 1 week.

**Table:** Stimulation culture media

Reagent	Final concentration	Amount
RPMI 1640 media	88%	9 mL
Heat inactivated FBS	10%	10 mL
L-Glutamine	1% (1 mM)	0.01 mL
Fungizone (Amphotericin B)	1% (2.5 μg/mL)	0.01 mL
**Total**		**10 mL**

Filter through 0.22 μm membrane filter. Store at 4°C for up to 1 week.

**Table:** Freezing media

Reagent	Final concentration	Amount
Heat inactivated FBS	90%	4.5 mL
DMSO	10%	0.5 mL
**Total**		**5 mL**

Store at 4°C for up to 1 week.

**Table:** Mass cytometry chemokine receptor cocktail

Antigen	Element + isotope Mass	Stock concentration (mg/mL)	Vol per 50 μL (μL)	+ 10%
CXCR3	Dy163	0.2	1	11
CCR7	Yb171∗	0.5	1	11
		MaxPar cell staining buffer vol	48	528

∗In-house conjugated antibodies. Volumes are calculated for 10 reactions + 10%. Prepare fresh, store at 4°C until use.

**Table:** Mass cytometry surface antibody cocktail

Antigen	Element + isotope Mass	Stock concentration (mg/mL)	Vol per 50 μL (μL)	+ 10%
CD45	Y89	0.2	1	11
CD206	Cd112∗	0.5	0.5	5.5
CD103	Nd145∗	0.5	0.5	5.5
CD33	Nd148∗	0.5	0.5	5.5
CD56	Sm149	0.2	0.25	2.75
CD274	Nd150∗	0.5	1	11
CD14	Eu151	0.2	1	11
CD163	Sm154	0.2	1	11
CD169	Gd158	0.2	0.5	5.5
CD11c	Tb159	0.2	1	11
CD66	Dy161∗	0.5	0.125	1.375
CD19	Dy164∗	0.5	0.25	2.75
CD40	Ho165	0.2	1	11
CD11b	Er167	0.2	1	11
CD32	Tm169	0.2	0.5	5.5
CD3	Er170	0.2	1	11
HLA-DR	Yb173∗	0.5	0.5	5.5
CD89	Yb176∗	0.5	1	11
CD16	Bi209	0.2	1	11
		MaxPar cell staining buffer vol	13.375	147.125

∗In-house conjugated antibodies. Volumes are calculated for 10 reactions + 10%. Prepare fresh, store at 4°C until use.

**Table:** Mass cytometry intra-cellular antibody cocktail

Antigen	Element + isotope Mass	Stock concentration (mg/mL)	Vol per 50 μL (μL)	+ 10%
TNF	Nd146	0.2	1	11
IL1ß	Sm152∗	0.5	0.5	5.5
IL-6	Gd156	0.2	0.5	5.5
		MaxPar PERM S buffer vol	2	22

∗In-house conjugated antibodies. Volumes are calculated for 10 reactions + 10%. Prepare fresh, store at 4°C until use.

### Mass cytometer setup

On day of acquisition, ensure that the CyTOF instrument passes quality control checks before thawing cells in batches.^5^ Ensure that the plasma warms up for a minimum of 30 min before performing auto-tuning. Each mass cytometry instrument will have different sensitivity and optimum dual counts values for metals present in the tuning solution (i.e., Tb159). Overall, we recommend to check detector voltage, oxide ratios (should be <3%) and CVs and instrument manuals should be the reference. For example, the CyTOF 2 instrument used for this protocol set a minimum of Tb159 dual counts of 650 000 following auto-tuning as well as an assessment of historic tuning performance to ensure maximum isotope signal detection prior to running samples. At a minimum, we recommend that Iodine and Barium 129–139 channels are selected in the acquisition template in addition to labeled antibody channels to monitor environmental/sample contamination from these elements. We recommend thawing a maximum of 6 vials at the time. Although the cells are fixed, they can degrade if kept pelleted in water for prolonged periods.

## Step-by-step method details

### BAL cell thawing and stimulation

**Timing: 7 h 30 min (includes 6 h stimulation time)**

**Timing: 1 h (for step 1)**

**Timing: 6 h 30 min (for step 2)**

This section describes the thawing and optimized culture conditions of cryopreserved BAL cells stimulated with mycobacterial antigens BCG and Mtb lysate.

**Figure 1:**
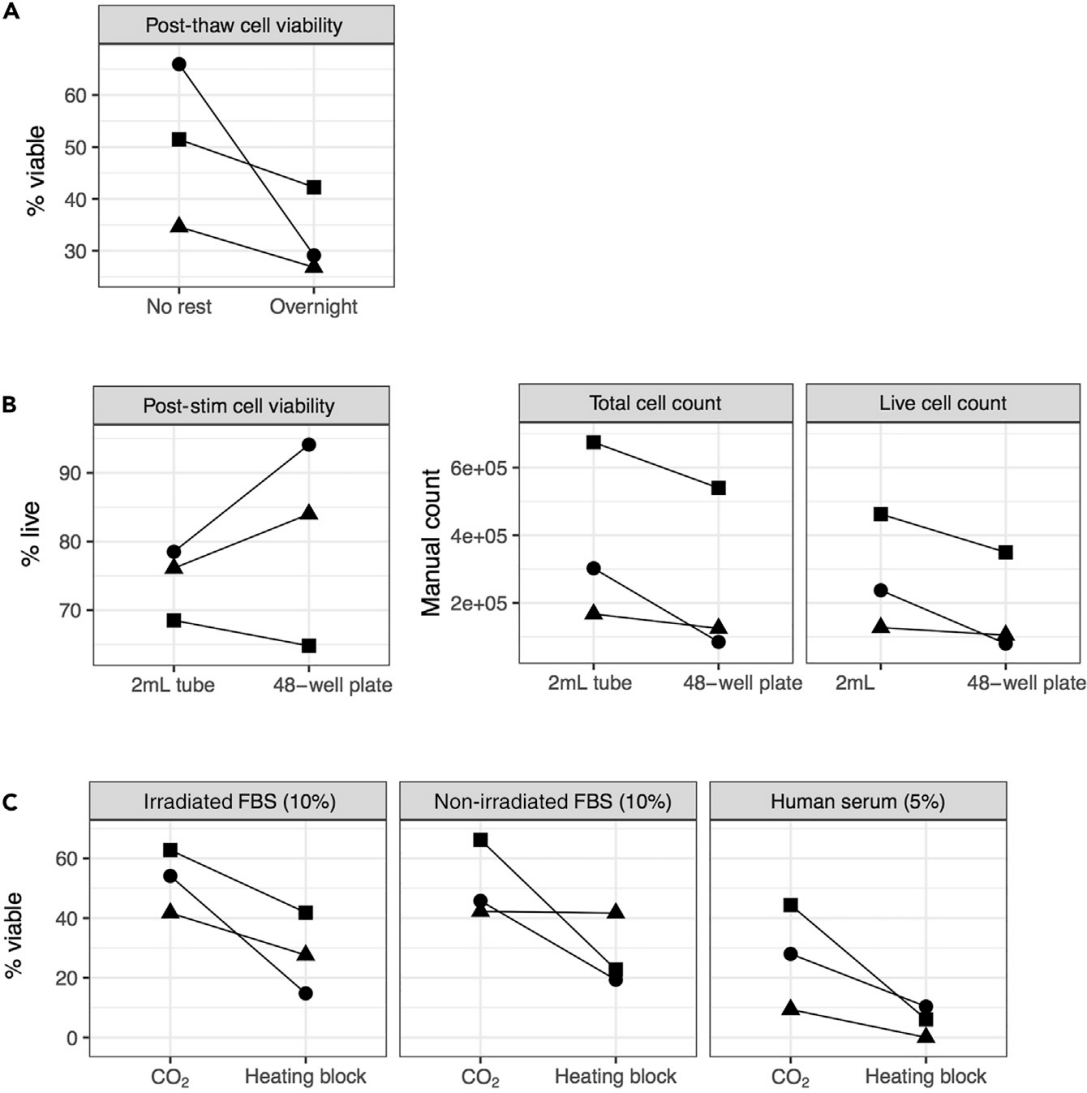
Optimized cell culture conditions for improved cell viability and recovery of BAL cells (A) Cell viability was measured in 3 independent BAL samples within an hour of thawing (no rest) vs. BAL samples rested 14–16 h in a 5% CO_2_ incubator at 37°C. Resting overnight decreased BAL cell viability compared to no rest. (B) Cell viability and recovery in 3 independent unstimulated BAL samples was compared after 6 h incubation in a 5% CO_2_ incubator at 37°C between cells in 2 mL tubes vs. 48 well plates. Cell viability was improved in 2/3 samples in plates while cell recovery was lower in 3/3 samples incubated in plates compared to tubes. (C) Cell viability was measured for 3 BAL samples cultured for 6 h in RPMI media with different serum supplementation (irradiated vs. non-irradiated FBS vs. human serum) and incubator conditions. Samples incubated in 2 mL tubes in a 5% CO_2_ incubator at 37°C (screw lids loosened) were more viable than samples incubated in 2 mL tubes in a heating block at 37°C (screw lids tightened).

**Figure 2:**
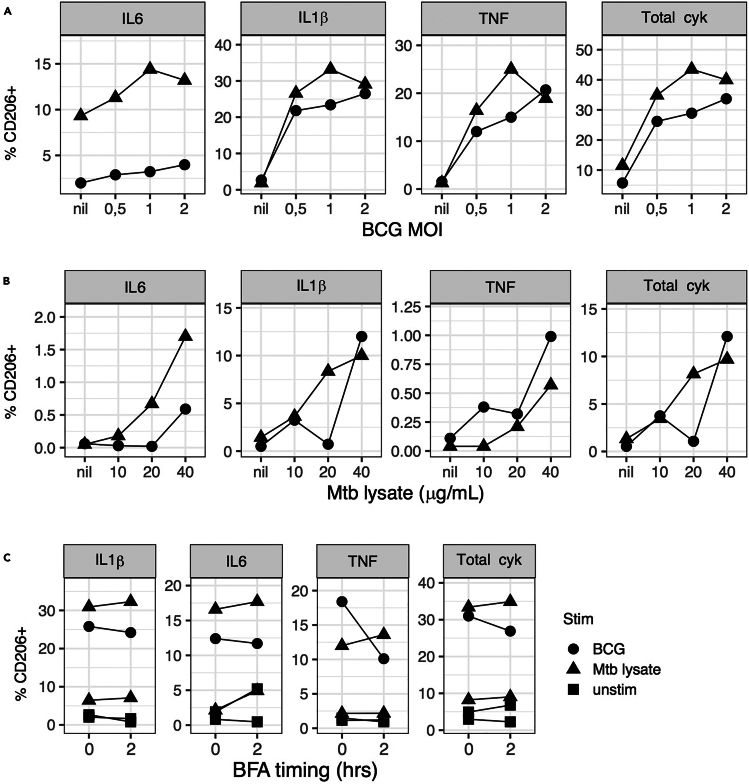
Optimized antigen concentrations and BFA timing for cytokine detection in BAL cells Frequencies of gated CD206+ cells producing cytokine (y axis) were measured after BAL samples were incubated for 6 h at increasing concentrations of mycobacterial antigens BCG (A) or Mtb lysate (B) ) in two different donors (circle and square symbols). MOI (multiplicity of infection) was calculated as the average ratio of live BCG CFUs per live BAL cell. (C) Frequencies of gated CD206+ cells producing cytokine were measured after BFA addition to stimulation culture (0 vs. 2 h).

***Note:*** In our experience, improved cell viability is observed when cells are not rested after thawing (Figure 1A) and are incubated in 2 mL Sarstedt tubes in a CO_2_ incubator with screw caps loosened (Figures 1B and 1C). RPMI media supplemented with fetal bovine serum (FBS) should be pre-filtered and pre-warmed in a 37°C water bath as described in the materials and equipment section. Cells can be cultured in either RPMI media supplemented with either irradiated or non-irradiated FBS which improve viability compared to human serum (Figure 1C). The antigen concentrations recommended here (10^6^ BCG CFU for 10^6^ live BAL cells and 30 μg/mL M.tb lysate) was selected to detect the maximum total proinflammatory signal in BAL cells after 6 h of culture (Figures 2A and 2B). If other antigens are used, their concentration should be titrated. BFA can be added at the start of cell culture with no loss of cytokine signal for alveolar macrophages stimulated with mycobacterial antigens (Figure 2C). The timing estimates for each section are for 3 individual BAL samples with 3 stimulation conditions (unstimulated, Mtb lysate and BCG) and 1 PBMC control with 2 stimulation conditions (unstimulated and Mtb lysate). For logistical reasons, cell staining is recommended on day 3, and the antigen stimulation reaction can be stopped by placing cells at 4°C for 14–16 h.

***Optional:*** PBMCs from a single donor can be run in parallel with each BAL cell experiment as a control for batch variation.

**CRITICAL:** All preparations of media and cells should be carried out in a biosafety cabinet to ensure sterility and minimize exposure to environmental Barium. Iodine is another common contaminant detected in mass cytometry therefore iodine-based cleaning reagents should not be used to disinfect surfaces.

1. Cell thawing.
  a. Set water bath to 37°C.
  b. Pre-fill labeled 50 mL conical tubes with 10 mL of pre-warmed (37°C) thawing media (refer to materials and equipment section for preparation).
  c. Thaw cells in water bath (stable at 37°C) until a small icy pellet of cells forms.
  d. Transfer vials to biosafety cabinet. Wipe vials with 70% ethanol before opening.
  e. Aliquot 1 mL of pre-warmed thawing media dropwise to each cryovial using a sterile Pasteur pipette.
  f. Transfer the cells into the pre-filled 50 mL conical tubes containing 10 mL of thaw media.
  g. Wash out cryovials with 1 mL of thaw media and transfer contents to 50 mL tube.
  h. Top up 50 mL conical tubes up to 20 mL of warm thaw media for a total volume of 20 mL.
  i. Centrifuge cells at 300 × *g* for 10 min at 18°C–24°C .
  j. Decant media and resuspend pellet by gently tapping conical tubes together.
  k. Top with 20 mL of warm thaw media and repeat steps i and j above.
  l. Add 5 mL of warm media to each conical tube. Ensure even cell suspension by pipetting the cell suspension up and down gently with a 5 mL serological pipette.
  m. Add 5 μL of thawed DNase I stock to cell suspension to obtain a final concentration of 24K units/mL and incubate for 20 min at 37°C.
  n. While cells are incubating, perform cell live cell count by trypan blue staining.
  o. Centrifuge cells at 300 × *g* for 10 min at 18°C–24°C.
  p. Decant media and resuspend pellet by gently tapping conical tubes together.
  q. Top up to 20 mL with plain warm RPMI 1640 media (antibiotic-free).
  r. Repeat steps o and p above.
  s. Resuspend cells to a live cell concentration of 10 × 10^6^ cells/mL in stimulation media (refer to materials and equipment section for preparation).
2. Cell stimulation.
  ***Note:*** Cell culture conditions are optimized for 1 × 10^6^ live cells in a final volume of 500 μL in stimulation media (refer to materials and equipment section for preparation). We recommend a minimum final volume of 250 μL. In this case, antigen and media volumes can be scaled down accordingly.
  a. Distribute 100 μL of cells (containing 1 × 10^6^ live cells) resuspended in stimulation media to each labeled sterile 2 mL polypropylene tubes.
  b. **Tube 1:** To each unstimulated condition (negative control), add 380 μL of stimulation media.
  c. **Tube 2:** To each BCG stimulated condition, prepare working stock on the day of stimulation as detailed in the reagent preparation section.
    i. Add 280 μL of stimulation media + 100 μL of BCG resuspended in stimulation media (10 × 10^6^ CFU/mL) to each BCG tube to obtain a final concentration of 10^6^ CFU for 10^6^ live BAL cells in 500 μL.
      ***Note:*** There is batch to batch variation in the number of BCG CFU per vial, where each lyophilized BCG vaccine (SSI) vial contains between 2–8 × 10^6^ CFU of BCG. The final MOI 1 is calculated using an estimated median vial concentration of 5 × 10^6^ CFUs.
  d. **Tube 3:** To the Mtb lysate stimulated condition, add 280 μL of stimulation media + 100 μL of pre-thawed Mtb lysate stock (150 μg/mL) to give a final concentration of 30 μg/mL.
  e. Add 20 μL of 250 μg/mL brefeldin A to each tube to give a final concentration of 10 μg/mL.
  f. Vortex tubes briefly and loosen 2 mL tube lids before placing in a 37°C, 5% CO_2_ incubator for 6 h.
  g. At end of 6 h incubation, screw the 2 mL tube lids tightly to avoid contamination and place at 4°C for 14–16 h.
    **CRITICAL:** BAL cells should be incubated in 37°C, 5% CO_2_ with 2 mL tube lids loosened as opposed to heating block incubators as cell viabilities are impacted by changes to pH in culture media (see Figure 1C).

### BAL cell staining for mass cytometry

**Timing: 7 h**

**Timing: 30 min (for step 3)**

**Timing: 4 h 30 min (for step 4)**

**Timing: 45 min (includes 15 min washing steps) (for step 4a)**

**Timing: 55 min (includes 25 min washing steps) (for step 4b)**

**Timing: 55 min (includes 25 min washing steps) (for step 4c)**

**Timing: 40 min (includes 15 min washing steps) (for step 4d)**

**Timing: 55 min (includes 15 min washing steps) (for step 4e)**

**Timing: 14–16 h at 4°C or 1 h at 18°C–24°C (for step 4f)**

This section describes the harvesting of BAL cells after antigen stimulation and staining with metal-conjugated antibodies for surface and intra-cellular markers.

**CRITICAL:** Supernatants should be aspirated by sterile Pasteur pipette carefully without dislodging the cell pellet leaving approximately 50 μL in each tube. To avoid cross-contamination, use a single pipette to aspirate from tubes in the following order for one sample donor: 1) unstained, 2) unstimulated, and 3) stimulated. Washing steps should not be skipped as they reduce background signal, measurement error and wear and tear on the mass cytometer’s detector.

3. Cell harvesting.
  ***Optional:*** (But recommended for BAL cells) Include unstained control (stained only with intercalator-Iridium 191/193). These cells should be run in parallel with stained tubes with the same wash buffer and fixation steps as experimental tubes.
  a. Label 5 mL snap cap FACS tubes with sample ID and conditions.
  b. Remove Sarstedt stimulation tubes from 4°C and place on ice.
  c. Add 1.2 mL of cold 1 x barium-free PBS 2.5 mM EDTA to each tube.
  d. With a sterile pipette mix very carefully to ensure tube contents do not spill out.
  e. Place tubes on ice for 15 min.
  f. Carefully transfer the cell mix from each tube to corresponding labeled 5 mL snap cap FACS tubes.
  g. Wash out stimulation tubes with 1 mL of cold 1 x PBS before transferring to the corresponding labeled 5 mL snap cap FACS tube.
  h. Centrifuge cells at 300 × *g* for 10 min at 18°C–24°C .
  i. Aspirate the supernatant with sterile Pasteur pipette.
  j. Add 2 mL of 1 x barium-free PBS and repeat centrifugation and aspiration steps.
  k. Tap tubes to resuspend cells.
4. Cell staining.
  a. Viability staining.
    ***Note:*** Cell-ID Intercalator-Rh must be pre-titrated for optimal staining. Typically the range is between 1:500–1:1000 in 1 mL of PBS which translates to a working concentration of 0.5–1 μM.
    i. Prepare the titrated dilution Cell-ID Intercalator-Rh 500 μM (Rh103) solution in 1 x barium-free PBS.
    ii. Add 1 mL of Rh103 solution to each experimental tube (except for the unstained control tube).
    iii. Mix well to ensure even cell suspension using a P1000 pipette with filtered tips.
    iv. Incubate for 20 min at 18°C–24°C.
    v. Add 2 mL of MaxPar cell staining buffer to all tubes.
    vi. Centrifuge at 300 × *g* for 10 min at 18°C–24°C.
    vii. Aspirate the supernatant and repeat washing steps (v and vi) for a total of 2 washes.
  b. Chemokine receptor staining.
    i. Aspirate the supernatant with sterile pipette to leave ∼50 μL of buffer.
    ii. Tap tubes to resuspend cells.
    iii. Add 50 μL of chemokine receptor cocktail to each experimental tube (except for the unstained control tube).
    iv. Mix well to ensure even cell suspension using a P100 pipette with filtered tips.
    v. Incubate for 30 min in a 37°C 5% CO_2_ incubator.
    vi. Add 2 mL of MaxPar cell staining buffer to all tubes.
    vii. Centrifuge at 300 × *g* for 10 min at 18°C–24°C.
    viii. Aspirate the supernatant and repeat washing steps (vi and vii) for a total of 2 washes.
  c. Surface staining.
    i. Aspirate the supernatant with sterile pipette to leave ∼50 μL of buffer.
    ii. Tap tubes to resuspend cells.
    iii. Add 50 μL of surface cocktail (prepared in MaxPar staining buffer) to each experimental tube (except for the unstained control tube).
    iv. Mix well to ensure even cell suspension using a P100 pipette with filtered tips (use new tips for each tube).
    v. Incubate for 30 min at 18°C–24°C.
    vi. Add 2 mL of MaxPar cell staining buffer to all tubes.
    vii. Centrifuge at 300 × *g* for 10 min at 18°C–24°C.
    viii. Aspirate the supernatant and repeat washing steps (vi and vii) for a total of 2 washes.
  d. Fixation and permeabilization.
    i. Aspirate the supernatant with sterile pipette to leave ∼50 μL of buffer.
    ii. Gently tap tubes to resuspend cells.
    iii. Add 1 mL of 1 x MaxPar Fix I (diluted in 1 x barium-free PBS) to each tube.
    iv. Mix well to ensure even cell suspension using a P1000 pipette with filtered tips (use new tips for each tube).
    v. Incubate for 20 min at 18°C–24°C .
    vi. Add 2 mL of MaxPar Perm S buffer to all tubes.
    vii. Centrifuge at 800 × *g* for 5 min at 18°C–24°C .
    viii. Repeat permeabilization steps (vi-vii) for a total of 2 washes in Perm S buffer.
  e. Intracellular cytokine staining.
    i. Aspirate last wash leaving approximately 50 μL of buffer.
    ii. Add 50 μL of intracellular cytokine cocktail (prepared in MaxPar Perm S buffer) to each tube (except for the unstained control tube).
    iii. Mix well to ensure even cell suspension using a P100 pipette with filtered tips (use new tips for each tube).
    iv. Incubate for 30 min at 18°C–24°C .
    v. Add 2 mL of MaxPar cell staining buffer to all tubes.
    vi. Centrifuge at 800 × *g* for 5 min at 18°C–24°C.
    vii. Aspirate the supernatant and repeat washing step for a total of 2 washes.
  f. Cell-ID Intercalator-Iridium (Ir 191/193) staining.
    i. Aspirate the supernatant with sterile pipette to leave ∼50 μL of buffer.
    ii. Tap tubes to resuspend cells.
    iii. Prepare 1:1000 dilution of Ir 191/193 in MaxPar Fix and Perm buffer.
    iv. Add 0,5 mL of Ir 191/193 diluted in MaxPar Fix and Perm buffer to all tubes.
    v. Vortex and keep at 4°C overnight or incubate for 1 h at 18°C–24°C .

### Long-term storage of stained cells

**Timing: 25 min (for step 5)**

**Timing: 45 min (for step 6)**

**Timing: 1–6 h (for step 7)**

This section describes the steps to cryopreserve stained BAL cells, sample thawing and acquisition at the mass cytometer. In our experience these cells can be stored to 6 weeks without a loss in staining quality. Longer storage times are possible but need to be validated.

5. Removing excess Cell-ID Intercalator-Iridium (Ir 191/193) and cryopreservation (optional).
  a. Add 2 mL of MaxPar cell staining buffer to wash off Ir 191/193.
  b. Centrifuge at 800 × *g* for 5 min at 18°C–24°C.
  c. Aspirate the supernatant with sterile pipette and repeat wash step with 3 mL of MaxPar cell staining buffer for a total of 2 washes.
  d. Aspirate the supernatant with a sterile pipette to leave ∼50 μL of buffer.
  e. Tap tubes to resuspend cells.
  f. Optional cryopreservation:
    i. Add 500 μL of cold 10% DMSO in 0.22 μm filtered FBS to each tube.
    ii. Transfer tube contents into labeled cryovials.
    iii. Transfer cryovials into Mr. Frosty Freezing containers and store at −80°C for 14–24 h.
    iv. For long term storage, transfer samples to liquid nitrogen.
6. Thawing of stained samples.
  a. Remove samples from −80°C and allow to thaw to 18°C–24°C.
  b. Add 1 mL of MaxPar cell staining buffer to cryovials.
  c. With individual sterile pipette, mix well and transfer contents to 5 mL snap-cap tubes.
  d. Wash out cryovials with an additional 1 mL of MaxPar cell staining buffer and transfer contents to 5 mL snap-cap tubes.
  e. Centrifuge at 800 × *g* for 5 min at 18°C–24°C .
  f. Aspirate the supernatant with sterile pipette to leave ∼50 μL of buffer.
  g. Tap tubes to resuspend cells.
  h. Repeat wash steps with 2 mL of MaxPar cell staining buffer for a total of 2 washes.
  i. Aspirate the supernatant and add 2 mL of Milli-Q water.
  j. Centrifuge at 800 × *g* for 5 min at 18°C–24°C .
  k. Aspirate the supernatant with sterile pipette.
  l. Repeat wash steps with 2 mL of Milli Q water for a total of 2 washes.
  m. Keep cells pelleted in ∼50 μL of Milli Q water and place on ice.
7. Sample acquisition at the mass cytometer.
  a. Calculate the amount of diluted EQ Four Element bead suspension required for acquisition on the day (minimum 1 mL per sample recommended for BAL cells).
  b. Prepare 1:10 dilution of EQ Four Element calibration beads in Milli Q water.
  c. Resuspend 1^st^ sample in 1 mL of diluted EQ Four Element bead suspension.
  d. Using a sterile 1 mL syringe, draw 200 μL of sample and inject into the instrument loop.
  e. Using a fresh sterile syringe, draw 400 μL of Milli Q water and inject into the same loop in d above.
  f. In the acquisition window, monitor the event rate and Barium channel of the first events to reach the detector to determine if the sample requires further dilution.
  g. Wash sample loops between acquisition with 500 μL wash solution or 2% Hydrochloric acid solution followed by 500 μL water.

## Expected outcomes

**Figure 3:**
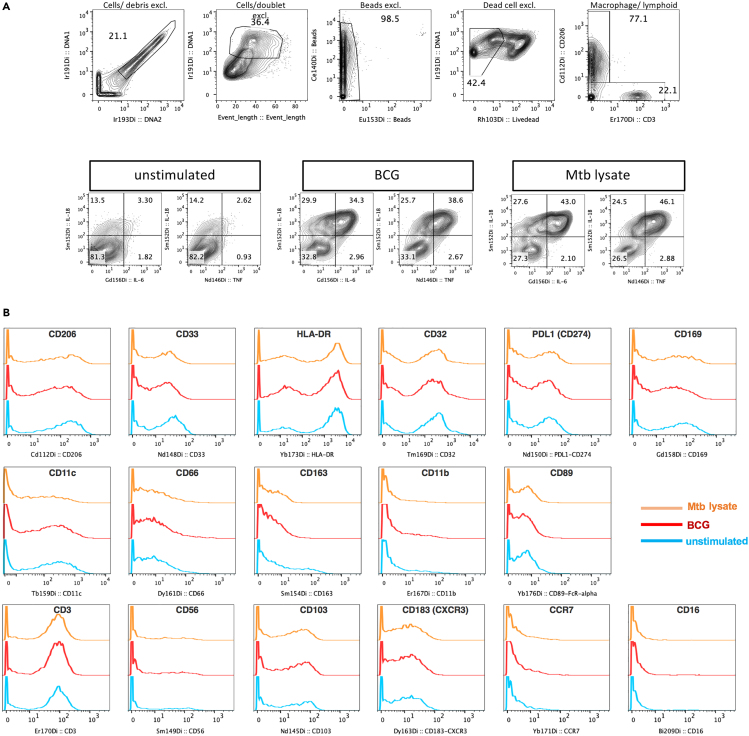
Expected outcomes for mycobacterial antigen responses and marker expression by BAL cells (A) Basic gating strategy to define alveolar macrophages (upper panels) and cytokine responses to mycobacterial antigens (lower panels). Firstly, intact cells were identified based on DNA intercalator Ir191/Ir193 positive staining which excluded debris. Removal of doublets by gating out high event length and further debris exclusion by gating on Ir191 positive cells with a heatmap overlay function set to CD45 expression (to define the lower boundary of Ir191 such that only CD45 events were included; not shown on contour plot). Four element calibration beads that are controls for normalization were gated out while live cells were defined as negative for Rh103. Macrophage and lymphocyte lineages were gated on CD206+CD3- and CD206- events respectively. (B) Histograms show the expected distribution in staining intensity (x-axes) for myeloid and lymphoid markers in BAL cells under different stimulation conditions (blue: unstimulated, red: BCG-stimulated, orange: Mtb lysate-stimulated. Marker expression is shown for live CD45+ events.

**Figure 4:**
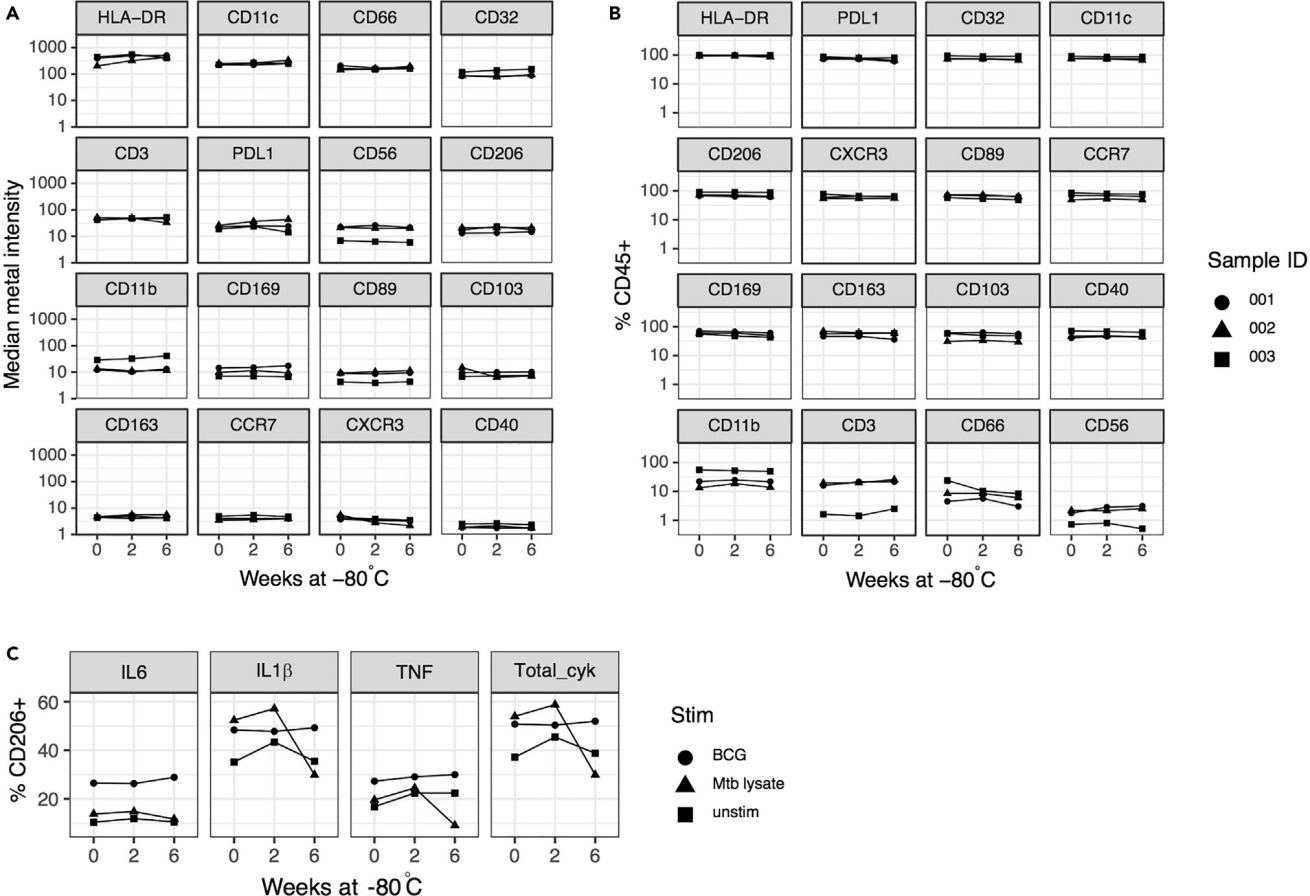
Stability of stained BAL samples after freezing Panel markers were measured in 3 independent BAL-stained samples without freezing (0 weeks) and after 2 and 6 weeks at −80°C for (A) median metal intensity, (B) frequency of live CD45+ expression and (C) frequency of CD206+ cells expressing cytokines. Overall, staining intensities, frequency of marker expression in cryopreserved samples was comparable to fresh samples.

The expected range of cytokine-producing myeloid cells stimulated with mycobacterial antigens is between 10% and 50% of live cells (see Figure 3A). Many markers in this panel exhibit a bi-modal distribution in BAL cells with moderate to excellent resolution between positive and negative signal (see Figure 3B). CD16 and CD56 expression in the BAL is rare in our experience therefore the inclusion of a control that includes a basic cocktail of antibodies to identify live leukocytes and excluding the marker of interest is recommended during panel validation to determine true signal.^6^ We demonstrate that the signal and quality of stained fresh BAL cell is comparable to stained cryopreserved BAL cells for up to 6 weeks (see Figure 4) allowing for batch analyses or the flexibility to freeze cells during instrument downtime as it has been previously shown for PBMCs (tested up to 1 month).^7^

## Quantification and statistical analysis

Raw fcs files require pre-processing prior to manual gating or any high dimensionality analyses. The pre-processing involves bead-based normalization to account for the loss of signal over the time of acquisition^8^ when using a CYTOF2 or Hyperion (note: the latest version of the instrument allow for regular tuning along acquisition avoiding loss of signal, however bead normalization is still recommended). In this protocol, we used the Premessa package in R (Parker Institute for Cancer Immunotherapy, 2016) which is an implementation of the original MATLAB normalization software which is no longer in use. A full description for the installation requirements and use of this R package and the accompanying graphical user interface is described elsewhere.^9^ After FCS files are normalized, they can be imported into FlowJo or R for manual gating and/or the implementation of high dimensionality reduction algorithms such as tSNE, UMAP or FlowSOM. A basic gating strategy for this panel to quantify mycobacterial responses is provided (Figure 3A).

## Limitations

The major limitations of this protocol are the number of samples processed for stimulation, staining and throughput on the mass cytometer instrument. For reproducibility, cells should be stimulated at a consistent cell concentration between experiment batches and if using a manual cell counting method, will increase laboratory processing time on Day 1. In mass cytometry, washing steps require aspiration of buffers to ensure uniformity in the removal of residual interfering buffer components and to minimize cell loss in the workflow.^1^ However, compared to decanting, this is more labor-intensive and should be factored when considering the number of samples processed. During sample acquisition, two main factors affect throughput: 1) the sample concentration and 2) Barium signal in the sample and contaminating reagents. Both factors require sample dilution prior to acquisition which significantly extends acquisition time. Barium is common in dish washing soaps and therefore reagents should be stored in either plastic containers or glassware that has not been washed with laboratory soap.^3^ In our experience, Barium signal in BAL samples is more common from participants who are smokers or those exposed to burning carbon fuels.

## Troubleshooting

### Problem 1: Low cell yield/viability

Low cell numbers after sample acquisition can lead to spurious results when quantifying mycobacteria-reactive cells (related to steps 2 and 4).

### Potential solution

In our experience, cryopreserved BAL cells are susceptible to cell death in culture even after a few hours of stimulation. We recommend the following.

• Not resting prior to antigen stimulation and.
• Incubating cells in 2 mL tubes in a 5% CO_2_ incubator at 37°C with screw lids loosened (Figure 1).

The number of wash steps necessary in mass cytometry staining will inevitably reduce the number of cells that are loaded onto the mass cytometer.

• Ensure that the correct centrifugation settings are used and confirm that cells are pelleted prior to aspiration.
• Aspirate buffers carefully not to dislodge the cell pellet.

### Problem 2: Poor marker resolution

Sub-optimal separation between positive and negative signal may lead to inaccurate classification of true positives by manual gating and spuriously defined populations by unbiased clustering algorithms (related to step various steps).

### Potential solution

**Figure 5:**
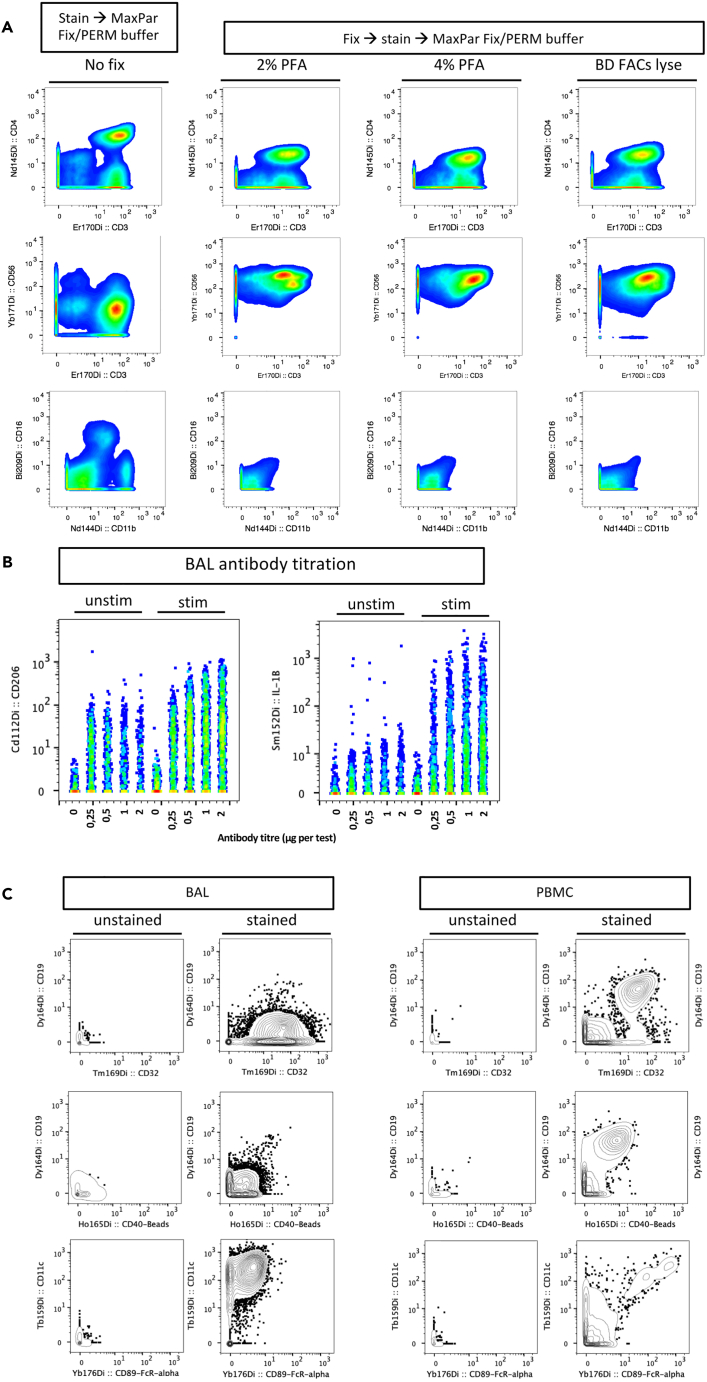
Optimizing antibody staining and resolution in BAL cells (A) Antibody staining was tested on fresh PBMCs followed by MaxPar Fix PERM incubation with Ir 191/193 (no fix) after fixation with 2% and 4% paraformaldehyde (PFA) or BD FACS lyse buffer followed by MaxPar Fix PERM incubation with Ir 191/193 for antibodies CD4 clone RPA-T4 (row 1), CD56 clone NCAM16.2 (row 2), CD16 clone 3G8 and CD11b clone ICRF44 (row 3). Staining after fixative treatment reduced the resolution of these antibody clones compared to fresh cells. (B) Titrations of surface marker CD206 and intra-cellular marker IL1ß on BAL cells highlights the importance of titrating markers on both stimulated and unstimulated cells. (C) BAL or PBMCs were stained with the full antibody cocktail (stained) and compared to unstained controls (Ir 191/193 staining only) to determine true positive signal for markers CD19 and CD32 (row 1), CD19 and CD40 (row 2), CD11c and CD89 (row 3).
• All antibodies should ideally be titrated on both stimulated and unstimulated cells as expression patterns may differ by stimulation (Figure 5B). For inducible intra-cellular markers (e.g., cytokine), the antigen of choice should be pre-titrated to ensure optimal separation (see Figure 2B).
• Including unstained controls during panel design that will help define true positive events for a marker of interest (see Figure 5C).
• Some markers may be poorly defined in BAL cells (e.g., CD16 or CD19) and should be validated on tissue types where their expression is more common (Figure 5C).
• In addition, if possible, titrate antibodies on more than one donor as expression may vary between individuals.

• Cells treated with fixative prior to surface antibody staining can lead to poor resolution. Ensure that the antibody clones used are compatible with fixative by testing unfixed cells (see Figure 5A).

### Problem 3: Cell events not counted on mass cytometer instrument

When the acquisition event rate is too high, the CyTOF software stops registering ion events (related to step 8).

### Potential solution

• Dilute the sample such that the event rate is at most 500 events per second.
• Barium or other non-specific metal signal will register as events on the mass cytometer detector and, if present in significant amounts, display a streak pattern on the rain plot accompanied by a high event rate. Dilute the sample such that the event rate is at most 500 events per second.
• Cell debris are common in BAL samples and may result in sample line clogging resulting in no ion events being registered in the masses/rain plot window. Samples resuspended in bead solution prior to acquisition should be filtered through a cell strainer snap cap into the Falcon 5 mL Round Bottom Polystyrene Tubes prior to sample loading. Ensure that sample lines are clear of debris by running wash solution and water between samples for at least 3 min each.

## Resource availability

### Lead contact

Further information and requests for resources and reagents should be directed to and will be fulfilled by the lead contact, Elisa Nemes (elisa.nemes@uct.ac.za).

### Technical contact

Questions about the technical specifics of performing the protocol should be directed to the technical contact, Agano Kiravu (agano.kiravu@gmail.com).

### Materials availability

This study did not generate new unique reagents.

### Data and code availability

Data and code are available from authors upon request.

## Consortia

Michele Tameris, Thomas Scriba, Arina Conradie, Fazlin Kafaar, Ilana C. van Rensburg, Gerhard Walzl, Stephanus Malherbe, Ayanda Shabangu, Keren Middelkoop. See Document S1 for consortium member affiliations.

## Acknowledgments

This work was funded by the US National Institutes of Health (BAA-NIAID-NIHAI201700104 and R01AI150850). L.C. is supported by a UCT doctoral award. We are grateful to the study participants. We thank Dr. Michele Tameris and the clinical team at the South African Tuberculosis Vaccine Initiative (SATVI) for participant recruitment. We also thank Prof Gerhard Wazl and Dr. Stephanus Malherbe who conducted the bronchoscopies as well as Devon Allies and Sean Vermeulen from the laboratory team for sample collection and processing. We acknowledge the contributions of the Stellenbosch University Immunology Research Group members I.C.v.R. and A.S.

## Author contributions

Conceptualization: E.N., V.R., and N.D. Data generation and analysis: A.K., L.C., and A.G. Writing – original draft: A.K. Funding acquisition: E.N.

## Declaration of interests

The authors declare no competing interests.

## Supplemental information

Document S1. Tables S1 and S2 and consortium member affiliations

## References

[1] Iyer A., Hamers A.A.J., Pillai A.B. (2022). CyTOF® for the Masses. Front. Immunol..

[2] Corporation F. Maxpar Panel Designer v2.01. https://www.standardbio.com/resources/panel-design.

[3] Leipold M.D., Newell E.W., Maecker H.T., Shaw A.C. (2015). Immunosenescence: Methods and Protocols.

[4] Shaw J.A., Meiring M., Allies D., Cruywagen L., Fisher T.L., Kasavan K., Roos K., Botha S.M., MacDonald C., Hiemstra A.M. (2023). Optimising the yield from bronchoalveolar lavage on human participants in infectious disease immunology research. Sci. Rep..

[5] Leipold M.D., Maecker H.T. (2012). Mass cytometry: protocol for daily tuning and running cell samples on a CyTOF mass cytometer. J. Vis. Exp..

[6] Au-Yeung A., Takahashi C., Mathews W.R., O’Gorman W.E., McGuire H.M., Ashhurst T.M. (2019). Mass Cytometry: Methods and Protocols.

[7] Sumatoh H.R., Teng K.W.W., Cheng Y., Newell E.W. (2017). Optimization of mass cytometry sample cryopreservation after staining. Cytometry A..

[8] Finck R., Simonds E.F., Jager A., Krishnaswamy S., Sachs K., Fantl W., Pe’er D., Nolan G.P., Bendall S.C. (2013). Normalization of mass cytometry data with bead standards. Cytometry A..

[9] Gheradini P.F. (2022). ParkerICI/ premessa. https://github.com/ParkerICI/premessa.

