# Supplementary material for: Protocol to phenotype and quantify mycobacteria-specific myeloid cells from human airways by mass cytometry
Source: STAR Protoc. 2024 Dec 13;5(4):103463. doi: 10.1016/j.xpro.2024.103463 (PMC11699420; doi:10.1016/j.xpro.2024.103463)
Supplement: Document S1. Tables S1 and S2 and consortium member affiliations [file mmc1.pdf]

Table S1: Recovery of in-house conjugated antibodies, related to reagent preparation

Antibody-conjugate	Unconjugated antibody stock input (μg)	Recovery of conjugated antibody (%)	Total yield of conjugated antibody (μg)
CD206 Cd112	200	89	178
CD103 145Nd*	200	55	110
CD33 148Nd*	200	56	112
CD274 150Nd*	300	68.5	205.5
IL-1B 152Sm	150	82	123
CCR7 171Yb*	300	62	186
CD89 176Yb*	300	41.5	124.5
CD66 161Dy	100	33	33
CD19 164Dy	200	46	92
HLA-DR 173Yb	200	33	66

*Average recovery from 2 independent runs

Table S2: Antibody titrations, related to reagent preparation

Isotope signal considerations				
**Target**	**Metal**	**Isotope**	**Signal into**	**Signal out of**
DNA	Ir	191		
DNA	Ir	193		
CD45	Y			
Live/dead	Rh			
CD206	Cd			
CD103	Nd	146Nd		161Dy 161Dy
TNF	Nd		145Nd, 148Nd, 163Dy 163Dy, 170Er	
CD33	Nd	146Nd, 149Nd		164Dy
CD56	Sm	154Sm	150Nd, 152Sm, 154Sm, 165Ho, 170Er	165Ho
PDL1	Nd	149Nd, 151Eu		
CD14	Eu		150Nd, 152Sm, 167Er	167Er
IL1b	Sm	149Nd, 151Eu, 154Sm		
CD163	Sm	149Sm	149Sm, 152Sm, 170Er	170Er
IL-6	Gd	158Gd		
CD169	Gd	156Gd, 159Tb	156Gd, 159Tb	
CD11c	Tb	158Gd	158Gd	
CD66	Dy	163Dy		
CXCR3	Dy	146Nd	161Dy, 164Dy	
CD19	Dy	163Dy, 165Ho		
CD40	Ho	149Sm	164Dy	
CD11b	Er	170Er, 151Eu	170Er	
CD32	Tm	170Er		
CD3	Er	167Er, 170Er, 154Sm	167Er, 170Er, 164Dy	
CCR7	Yb			
HLA-DR	Yb	156Gd	171Yb, 176Yb	
CD89	Yb	173Yb		
CD16	Bi			

Batched titration design				
**Titration experiment**	**Anchor markers**	**Marker**	**Expected cell subset expression**	**Sample type for titration**
1	CD45, CD3, CD16	CD19	B cells	PBMC
		CD11c	Myeloid cells	PBMC
		CD103	T cells	PBMC
		CD33	Myeloid cells	PBMC
		HLA-DR	Myleoid, T and B cells	PBMC
		CD11b	Myeloid cells	PBMC
		CD14	Monocytes	PBMC
		CD56	NK cells	PBMC
2	CD45, CD3	CD206	Alveolar macrophages	BAL
		CD89	Alveolar macrophages, DCs, B cells	BAL
		CD32	Alveolar macrophages, DCs	BAL
		CD163	Myeloid cells	BAL
		CD169	Myeloid cells	BAL
		CXCR3	Alveolar macrophages, DCs, T cells	BAL
		IL-1b	Alveolar macrophages	BAL

3	CD45, CD3, CD19, CD206	PD-L1	Alveolar macrophages, DCs	BAL
		CD89	Alveolar macrophages, DCs, B cells	BAL
		CD40	Alveolar macrophages, DCs, B cells	BAL
		CD66	Granulocytes	BAL
		CCR7	DCs, T cells	BAL

POI-BAL study group: Michele Tameris,[1] Thomas Scriba,[1] Arina Conradie,[1] Fazlin Kafaar,[1] Ilana C. van Rensburg,[2] Gerhard Walzl,[2] Stephanus Malherbe,[2] Ayanda Shabangu,[2] and Keren Middelkoop[3]

[1]South African Tuberculosis Vaccine Initiative, Institute of Infectious Disease and Molecular Medicine and Division of Immunology, Department of Pathology, University of Cape Town, Cape Town 7925, South Africa

[2]DSI-NRF Centre of Excellence for Biomedical Tuberculosis Research, South African Medical Research Council Centre for Tuberculosis Research, Division of Molecular Biology and Human Genetics, Faculty of Medicine and Health Sciences, Stellenbosch University, Cape Town, South Africa

[3]Desmond Tutu HIV Centre, Department of Medicine, Institute of Infectious Disease & Molecular Medicine, University of Cape Town, Cape Town, South Africa
